# Assessment of human immunity to A/H3N2 influenza subclade K during 2025 emergence

**DOI:** 10.1016/j.ebiom.2026.106332

**Published:** 2026-06-11

**Authors:** Ruth Harvey, Ceilidh Welsh, Alexander MP. Byrne, David Greenwood, Phoebe Stevenson-Leggett, Monica Galiano, Dalan Bailey, Yung-Wai Chan, Jacob Boateng, Hannah Wolmuth-Gordon, Heather Whitaker, Kevin E. Brown, Anna Jeffery-Smith, Alex Allen, Gayatri Amirthanlingam, Even Fossum, Elisabeth Lea Vikse, Andreas Rohringer, Olav Hungnes, Karoline Bragstad, Charles Swanton, Bryan Williams, Sonia Gandhi, Steve Gamblin, Edward J. Carr, Nicola S. Lewis, Emma C. Wall, Mary Y. Wu, Crick Serology Pipeline, Giulia Dowgier, Agnieszka Hobbs, Choon Ping Tan, Jarod Zvartau-Hind, Murad Miah, Odiesia Daley, Mauro Miranda, Philip Bawumia, Nicola O’Reilly, Chris Cheshire, Nicholas Wilson, Karen Ambrose, Amy Strange, Gavin Kelly, Svend Kjær, Legacy Investigators, Rupert CL. Beale, Padmasavee Papineni, Tumena Corrah, Richard Gilson

**Affiliations:** a Worldwide Influenza Centre, Francis Crick Institute, London, UK; b Francis Crick Institute, London, UK; c Viral & Immune Surveillance Platform, Francis Crick Institute, UK; d The Pirbright Institute, Ash Road, Pirbright, UK; e Immunisation and Vaccine Preventable Diseases Division, UK Health Security Agency, London, UK; f Statistics, Modelling and Economics Department, UK Health Security Agency, London, UK; g National influenza Centre, Oslo, Norway; h Division of Infection Control, Department of Virology, Norwegian Institute of Public Health, Oslo, Norway; i University College London, UK; j UCL Centre for Renal and Bladder Health, University College London, UK; k National Institute for Health Research Biomedical Research Centre, University College London Hospitals, London, UK; l Centre for Infection and Immunobiology, Blizard Institute, Queen Mary University of London, UK; m Royal Veterinary College, Royal College Street, London, UK

**Keywords:** Seasonal influenza, H3N2, Subclade K, Human serology, Antigenic cartography, Vaccine, Neutralising antibodies, Haemagglutination inhibition

## Abstract

**Background:**

Seasonal influenza causes significant morbidity and mortality annually. In 2025, the genetically divergent A/H3N2 K subclade (J.2.4.1) emerged with substantial haemagglutinin mutations. However, despite suggested antigenic escape, UK vaccine effectiveness estimates and epidemiological data demonstrated a relatively normal influenza season across 2025–26. We examined neutralising antibody responses in human cohorts to investigate existing and vaccine-induced immunity to K clade viruses.

**Methods:**

We characterised the antigenic relationships of a selection of A/H3N2 viruses spanning recent evolution including a subclade K virus using antigenic cartography, followed by serological antibody profiling of four human cohorts from the United Kingdom and Norway using microneutralisation (MN) and haemagglutination inhibition (HAI) assays.

**Findings:**

Antigenic cartography from single-infection ferret antisera suggests significant antigenic drift from the vaccine strains. MN and HAI titres from 243 individuals across 4 human cohorts (ages 1–105 years) were measured for comparison. The 2025/26 Northern Hemisphere seasonal inactivated egg-derived trivalent influenza vaccine (eTIV, with J.2 A/H3N2) significantly boosted MN and HAI titres against all A/H3N2 viruses tested, including a subclade K virus (p < 0.001). Furthermore, serological profiles of cohorts stratified by age groups (≤5, >5–≤15, >20–≤25, >25–<60, and ≥60) showed pre-existing reactivity against the emergent subclade K viruses, with minimal inter-age variation, suggesting there was not an immunity gap within particular age groups.

**Interpretation:**

The 2025/26 seasonal inactivated eTIV vaccine effectively boosted neutralising titres despite substantial genetic and antigenic drift. Human serological profiling should be included in risk assessments and continued surveillance.

**Funding:**

The Francis Crick Institute with core funding from Cancer Research UK, UK Medical Research Council, and Wellcome Trust; UK Research and Innovation and UK Medical Research Council; National Institute for Health Research University College London Hospitals Biomedical Research Centre; UK Health Security Agency; Norwegian Institute of Public Health.

**Research in context**

**Evidence before this study**

We searched the National Library of Medicine, EMBASE and MedRxiv from 1st January 2022 to 31st December 2025 for cohort studies or clinical trials that included evaluation of human antibody titres against Influenza H3N2 viruses, with a focus on K clade emergence. We used the search terms “Influenza AND (H3N2 OR Clade K)” AND “Antibody OR neutralisation OR Serum OR Vaccine” and set the filters to clinical cohorts or randomised clinical trials. We found two 2022 studies of antibody responses to seasonal quadrivalent influenza vaccine (QIV) containing H3N2 in humans, the first demonstrating broad induction of neutralising antibodies against drifted strains, the second a trial of an experimental intranasal H3N2 vaccine, demonstrating post-vaccine induction of 4-fold rise in titres across drifted viruses. A follow-on study from the same team shows that induction of neutralising antibodies in vaccine recipients subsequently protected them from experimental challenge with an antigenically distinct H3N2 virus. A systematic review and modelling study combined 15 clinical studies from 2017 to 2022 to develop a predictive model for susceptibility to H3N2 viruses, evaluated against prospective clinical data across three vaccine strains and geographical regions from 2022 to 3. The investigators reported that higher pre-vaccine HAI titres against emerging strains were most predictive of vaccine response and subsequent protection against emerging strains. Three publications and 2 preprints were found evaluating the emergence of the A/H3N2 subclade K viruses. Four studies suggest that antigenic escape of existing immunity and/or of the A/H3N2 vaccine strain may explain the unusual circulation patterns of subclade K viruses. One study provided early vaccine efficacy estimates during the 2025/26 Northern Hemisphere flu season, showing that the vaccine provides protection in the early period post-vaccination.

**Added value of this study**

We combined two methodologies and four cohort studies to report both HAI and neutralising antibodies to provide serological assessment of both seasonal inactivated eTIV boosting against emergent subclade K viruses as well as age-stratified background immunity in unvaccinated individuals. Our data show that despite significant antigenic drift of H3N2 subclade K, and concern regarding increased hospitalisations in the Southern Hemisphere, adults in the Northern European regions had detectable titres in both assays against all A/H3N2 viruses tested. Furthermore, the seasonal 2025 inactivated eTIV was able to boost neutralisation titres, reflecting the epidemiological data for the 2025–26 influenza season, including vaccine efficacy estimates.

**Implications of all the available evidence**

Assessments of pre-existing and vaccine-induced protective immunity in human populations to emerging antigenically drifted viruses can be conducted in near real-time and are potentially an important strategy for risk assessment and mitigation against future emergent Influenza viruses.

## Introduction

Influenza viruses circulate globally as seasonal influenza in humans–an acute respiratory infection that causes illness which ranges from mild to severe, sometimes resulting in hospitalisation and even death.^1^^,^^2^ Influenza viruses are antigenically and genetically variable and continually co-circulate and evolve over time. Periodically, new genetic groups of viruses emerge, known as clades and subsequently as subclades.^3^ Their genetic changes can result in antigenically distinct viruses, which are less recognised by prior immunity in the human population or are demonstrably distinct in *in-vitro* assessments of potential immunological cross-recognition by vaccine. In August 2025, a rapid rise in detections of A/H3N2 influenza viruses from the K subclade was reported from the Southern Hemisphere seasonal influenza surveillance.^4^ Whilst not unprecedented, these subclade K viruses have coincided with both a prolonged influenza season in some Southern Hemisphere countries and a notable earlier start to influenza epidemics in Northern Hemisphere countries, particularly in the United Kingdom, Japan and Norway.

Despite these early signs, and evidence showing that sera from ferrets infected with viruses representing Northern Hemisphere vaccine strains had low reactivity against subclade K viruses,^5^ subsequent epidemiology^6^ and early vaccine effectiveness estimates within expected ranges in the UK did not support a significant immune escape.^7^

Here, we characterise the antigenic drift along an evolutionary time course, from the Northern Hemisphere 2025–26 vaccine composition recommendation for A/H3N2 through to the emergence of the K subclade using antigenic cartography, followed by the profiling of population-level pre-existing and vaccine-induced A/H3N2 antibody immunity to risk assess actual antigenic escape in human hosts. We use age-stratified cohorts under serological surveillance from the UK and Norway to understand whether the rapid rise of the K subclade was due to an immunity gap, and we assess the effect of existing vaccine in providing antibody titres at a level expected to be protective.

## Methods

### Clinical cohorts

We analysed biobanked samples and metadata from three UK-based, and one Norwegian prospective cohort studies of individuals who consented to be under serological surveillance for respiratory viruses including influenza. We used samples collected prior to, and/or following seasonal trivalent influenza vaccination for the 2025/26 season where available. Where post-vaccine samples were not available, we analysed available samples at the most recent time-point in spring and summer of 2025 prior to seasonal circulation of A/H3N2 subclade K viruses to estimate population seroreactivity against this strain. We further stratified participants across the cohorts by age group to compare baseline antibody reactivity in individuals ≤5, >5–≤15, >20–≤25, >25–<60, and ≥60 years of age.

#### COVID-19 vaccine responses after extended immunisation schedules (CONSENSUS) audit

The CONSENSUS audit was set up by the UK Health Security Agency (UKHSA, formerly Public Health England) in 2021 to measure and compare the antibody levels of individuals receiving COVID-19 vaccines following the extended primary schedule.^8^ At the start of the audit, immunocompetent adults aged 50 years or above residing in London were recruited through General Practitioners. The CONSENSUS audit has been extended to monitor the immune responses of the participants following subsequent biannual COVID-19 vaccination campaigns.

All CONSENSUS participants were invited to be sampled to understand their immunological responses following the 2025 COVID-19 spring booster. Sera from 73 participants who received the 2024/25 Northern Hemisphere Influenza Vaccine, collected between May and August 2025, were included in this study.

#### Worldwide Influenza Centre (WIC) UK cohort

A cohort of healthy, influenza vaccinated adults under longitudinal WHO Influenza serological surveillance to inform vaccine strain selection decisions. Samples from 25 individuals were collected pre- and post-vaccination with the 2025/26 Northern Hemisphere inactivated eTIV and analysed as part of this study.

#### Legacy cohort

The Legacy study (NCT04750356) is a prospective observational cohort, established in January 2021 of adults undergoing PCR-based surveillance for SARS-CoV-2 and other respiratory viruses, including influenza, across four institutions in northwest London. Extensive descriptions of the cohort can be found in our prior reports.^9^, ^10^, ^11^ Participants were recruited to Legacy from January 2021, inclusion criteria were broad (adults employed by an institution using the Crick-COVID PCR testing pipeline and had undergone testing for SARS-CoV-2), exclusion criteria were limited to ability to provide written informed consent and willingness for longitudinal follow up and ongoing serological surveillance. Participants are followed up every six months. At each study visit, individuals gave details on any recent infection episodes, vaccination doses, and had blood drawn for serum for live-virus microneutralisation assays and SARS-CoV-2 anti-N IgG detection. Additional visits are conducted pre/post vaccination where possible and following a self-reported infection episode. Samples from 34 adults under the age of 25 were analysed as part of this study.

#### Norwegian Institute of Publica Health (NIPH) cohort

Serum samples from 21 healthy adults were collected 21–29 days (median of 23 days) after immunisation with inactivated eTIV for the 2025/26 season and analysed as part of this study.

Anonymised residual sera are collected annually for national seroepidemiology studies. Sera included for this study were collected in August 2025 from medical laboratories across Norway as previously described.^12^ In total, 90 sera were selected for analysis by HAI and low-throughput MN against A/Croatia/10136RV/2023, A/District of Columbia/27/2023 A and A/Norway/8765/2025. Twenty-five sera were selected from the age group 0–4 years old, 30 sera from 5 to 14 years old age group and 35 sera from the 70+ years old age group.

### Viruses

**Fig. 1:**
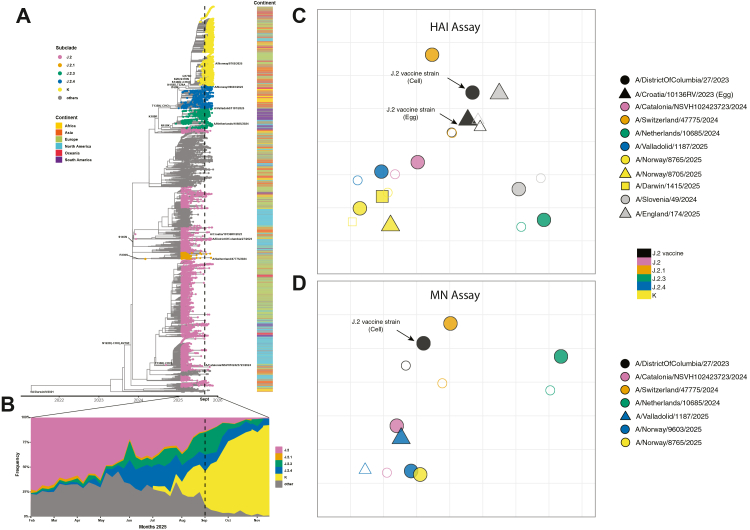
**Genetic, antigenic, and epidemiological comparison of emergent Influenza A/H3N2 strains.** (**A**) Time-resolved phylogenetic tree of contemporary A/H3N2 haemagglutinin (HA) sequences with collection dates from 1st February 2025 onwards, where tip shapes are coloured according to subclade and the continent of sample collection is shown. Key amino acid substitutions and reference viruses used in this study (Table S1) are annotated. (**B**) Timeline of frequencies of A/H3N2 subclades during 2025 based on all A/H3N2 full-HA sequences available on GISAID. (**C**) and (**D**) Antigenic cartography of A/H3N2 strains generated from haemagglutination inhibition (HAI; **C**) and microneutralisation (MN; **D**) assay data obtained with ferret antisera. In both C and D, strains are coloured by subclade and antigen shapes are used to distinguish strains of the same subclade. The J.2 vaccine strains recommended for the Northern Hemisphere 2025/26 season are indicated. Each square of the map grid denotes 1 antigenic unit or a 2-fold dilution in HAI or MN titres.

For the UK cohorts, we used eight A/H3N2 viruses from the evolutionary trajectory spanning the J.2. Northern Hemisphere A/H3N2 vaccine recommendations for the egg (A/Croatia/10136RV/2023) and the cell (A/DistrictOfColumbia/27/2023) components, through to selected genetic groups that either diverged (J.2.1, J.2.3), or resulted in the emerging subclade K (J.2.4, J.2.4.1) (Table S1 and Fig. 1). All viruses except A/Croatia/10136RV/2023 were propagated in MDCK-SIAT1 cells (CVCL_Z936, source Matrosovich lab, species authenticated and mycoplasma tested by the Francis Crick Cell Sciences STP) at 35 °C for 72 h.^8^ A/Croatia/10136RV/2023 was propagated in the allantoic cavity of 10-day-old embryonated hens' eggs at 35 °C for 48 h.^13^

The sequences of the viruses were confirmed after culture using a PCR amplification approach, followed by Illumina sequencing to ensure that the genetic characteristics were stable^14^

### Haemagglutination inhibition assays

Haemagglutination and HAI assays were performed according to standard methods^15^ using suspensions of guinea pig RBCs (1.0% v/v, Biological Services Colindale, UKHSA) in the presence of 20 nM oseltamivir carboxylate (Roche).^16^ All serum samples were pre-treated with receptor-destroying enzyme (RDE, Mast Group). Four haemagglutination units were used in all HAI assays.

### Microneutralisation assays

High-throughput live-virus microneutralisation assays were performed for influenza in MDCK-SIAT1 cells (CVCL_Z936) by adapting our existing approach for SARS-CoV-2 neutralisation and previously described in detail.^17^ Prior to use, all serum samples including controls were treated with receptor destroying enzyme (RDE, Mast Group) for 16 h at 37 °C at a ratio of 1:4 serum:RDE, followed by heat inactivation for 1 h at 56 °C. Serial dilution of RDE-treated samples were carried out in DMEM containing 1% Penicillin/Streptomycin and 25 mM HEPES pH 7.4 for use in the assay.

### Antigenic cartography

Antigen-serum antigenic maps were constructed using multidimensional scaling of MN and HAI data with the Racmacs R package [1.2.9].^18^^,^^19^ For MN data, seven strains of A/H3N2 and post-infection ferret sera raised against five of these viruses were analysed; whilst for HAI data, additional reference antigens and sera were used to demonstrate the antigenic diversity observed during the 2025/26 influenza season. Quality control included assessing residuals by comparing fitted and measured titres and evaluating uncertainty in antigen and sera positioning using map stress. To further evaluate map robustness, antigenic map uncertainty was assessed using 1000 bootstrap replicates of the titre data (Figure S2). In each replicate, titres were resampled and perturbed using the noisy bootstrap method to account for inherent measurement variability. Each bootstrap replicate was used to reconstruct the antigenic map, producing a distribution of possible coordinates for each antigen and serum. Confidence regions containing 68% of the bootstrap coordinates were calculated to represent and evaluate the stability of antigen position.

### Phylogenetic analyses

All (approximately 10,000) A/H3N2 full-length HA sequences available on GISAID with collection dates from 1 February 2025 onwards were downloaded for analysis, together with their metadata (download date 09/12/2025). Sequences were aligned with MAFFT and edited with BioEdit; a time-resolved Maximum likelihood tree was inferred with IQTree2 and Treetime.^20^ Sequences were classified by subclades with Nextclade (https://clades.nextstrain.org).^21^ Final tree and frequency timeline figures were produced using R scripts and Adobe Illustrator.

### Data analysis, and statistics

#### CONSENSUS

Data were stored in an access-controlled environment maintained by the UK Health Security Agency (UKHSA). All data were handled in compliance with UKHSA's policy.

#### WIC

Data were stored and managed in secure institutional systems in accordance with the General Data Protection Regulation (GDPR).

#### Legacy

Data used in this study were collected and managed using REDCap electronic data capture tools hosted at University College London (7).^22^^,^^23^ Data were imported to R from REDCap prior to analysis and integrated using R Chronogram as previously described.^24^

#### NIPH

Data were stored and managed in secure institutional systems in accordance with the General Data Protection Regulation (GDPR).

#### Data analysis and statistics

HAI and MN titres were associated with anonymised metadata in R (v 4.3.2)^25^ and analysed using the Tidyverse [2.0.0]^26^ suite of packages including ggplot2 [4.0.1].^27^ Discrete, interval-censored HAI titres and low-throughput MN were summarised by the geometric mean and geometric standard deviation. Continuous, left-censored MN titres reported as IC50 were summarised by the median and inter quartile range. To plot and analyse HAI and low-throughput MN titres, sera with no inhibition were re-coded as 2.5. To plot and analyse MN titres, IC50 values above the quantitative limit of detection of the assay (>40,000) were recoded as 80,000; IC50 values below the quantitative limit of the assay (IC50 < 40) but within the qualitative range were recoded as 10, and values below the qualitative range (i.e. no response observed) were recoded as 5. This recoding of censored values did not affect the statistical parameters considered (they fell outside the median and interquartile range), and analyses did not depend on absolute values because rank-based methods were used. Normality was assessed using the Shapiro–Wilk test; where persistent non-normality was observed (P < 0.05), non-parametric tests were applied. HAI and MN titres from paired sera were compared with Wilcoxon signed-rank tests as implemented in the rstatix [0.7.3] R package.^28^ Symmetry of the distributions of differences were assessed visually with histograms of paired fold-changes. HAI and MN titres from unpaired sera were compared pairwise between age groups using Wilcoxon rank-sum tests, with Holm–Bonferroni correction applied to control the family-wise error rate. To evaluate overall differences across the age groups the Jonckheere–Terpstra test for ordered alternatives was performed with age group as an ordinal factor. The 95% confidence intervals for fold-changes in HAI and MN were calculated from 1000 bootstraps generated with the infer [1.1.0] package.^29^ The 95% confidence intervals were obtained from the empirical 2.5th and 97.5th percentiles of the bootstrap distribution. Summary descriptions of the cohorts were generated using gtsummary [2.5.0].^30^ Continuous cohort summary data were reported as the median value and interquartile range (IQR), categorical data are reported as the count and percentage

### Ethics

The Legacy study was approved by London Camden and Kings Cross Health Research Authority (HRA) Research and Ethics committee (REC, reference 20/HRA/4717) IRAS number 286469 and is sponsored by University College London.

The WIC Cohort was approved by the Francis Crick Institute Internal Ethics Review Committee, registration number 2019FC5.

The CONSENSUS audit was approved by the UKHSA R&D Research Ethics and Governance Group (REGG reference number: NR0253).

Norwegian post-vaccination sera were collected with written, informed consent, and the study was approved by the REK (ref.nr. 157792). Collection of irreversibly anonymised residual sera in August 2025 has been approved by REK (ref.nr. 2009/1322) and does not require written, informed consent in accordance with the Norwegian Communicable Disease Control Act.

Unless stated otherwise, all human participants in the studies have given written, informed consent, and all studies comply with the 2024 Declaration of Helsinki ethical principles.

### Role of the funders

The funders of the study had no role in study design, data collection, data analysis, data interpretation, or writing of the manuscript. The corresponding authors had full access to all the data and the final responsibility to submit for publication.

## Results

To evaluate both pre-existing and vaccine-induced immunity against A/H3N2 viruses, we performed an assessment of the neutralising capability of sera drawn from across the lifecourse, as well as pre- and post-seasonal inactivate eTIV, in the UK and Norway, two Northern Hemisphere countries with early signals of subclade K dominance in autumn 2025. Participant demographics are shown in Table 1. To select the relevant “sentinel” viruses to encompass the diversity of circulating A/H3N2 viruses, we first performed a phylogenetic analysis of A/H3N2 over the past 2 years. Our analyses (Fig. 1A and B) showed that J.2 viruses bearing substitution K189R first circulated in early 2024 from which subclades J.2.3 (with N158K) and J.2.4 (with T135K and loss of N-glycosylation) emerged in the Northern Hemisphere autumn of 2024. These three mutations fall within 2 (A and B) of the 5 major antigenic sites (A–E) and changes in glycosylation can also lead to antibody evasion.^31^ Subclade K viruses were detected in June–July 2025 (with 7 additional mutations over J.2); by September they were circulating with a 30% frequency which increased to > 90% by November. To characterise the potential for cross-immunity we generated a panel of single-infection antisera in ferrets and used antigenic cartography to quantify the inter-relationships among the selected A/H3N2 strains from each of the genetic groups in an antigenic map, using either HAI assay or MN assay data (Fig. 1C and D). In both the HAI and MN maps, the J.2.3 group and the J.2.4/K viruses were antigenically distinct from the J.2 group, which included the cell and egg vaccine component representatives recommended for the 2025/26 Northern Hemisphere seasonal influenza vaccine. The J.2.3 and the J.2.4/K groups have evolved in two distinct antigenic trajectories, thus limiting the potential cross-protective responses between these co-circulating groups when assessed in either HAI or MN. These groups are approximately 3 antigenic units (Fig. 1C and D) or an 8-fold reduction in HAI and MN titres from the vaccine strains, so this would be considered on the threshold of loss of immunological recognition and a need to update vaccine composition.^23^

**Table 1:** Demographic details of included participants across evaluated cohorts.

Characteristic	CONSENSUS N = 73	Legacy N = 34	WIC N = 25	NIPH (Immunised) N = 21	NIPH (residual) N = 90
Sex (Female)	42 (58%)	27 (79%)	Not collected	15 (71%)	44 (49%)
Age (Median [IQR])	78 [55–99]	24 [23–25]	47 (28–73)	49 [44–54]	11 [4–75]
Age group (right closed)					
≤5		0 (0%)	0 (0%)	0 (0%)	29 (32%)
>5–≤15		0 (0%)	0 (0%)	0 (0%)	26 (29%)
>15–≤25		34 (100%)	0 (0%)	0 (0%)	0 (0%)
>25–≤60		0 (0%)	22 (88%)	18 (86%)	0 (0%)
>60		0 (0%)	3 (12%)	3 (14%)	35 (39%)
Age Group (left closed)					
≥55	73 (100%)				
Influenza vaccine (Northern Hemisphere 2024/5)	73 (100%) adjuvanted inactivated egg-derived QIV	10 (29%) Inactivated egg-derived QIV			
Influenza vaccine (Northern Hemisphere 2025/6)	0 (0%)	0 (0%)	25 (100%) Inactivated egg-derived TIV	21 (100%) Inactivated egg-derived TIV	
sampling relative to influenza vaccine (northern hemisphere 2025/6)	Pre	Pre	Pre/Post (14–40 days, median 20 days)	Post (21–29 days, median 23 days)	Pre

### Serological antibody profiling of cohort responses to recent A/H3N2 viruses: pre- and post-vaccination

**Fig. 2:**
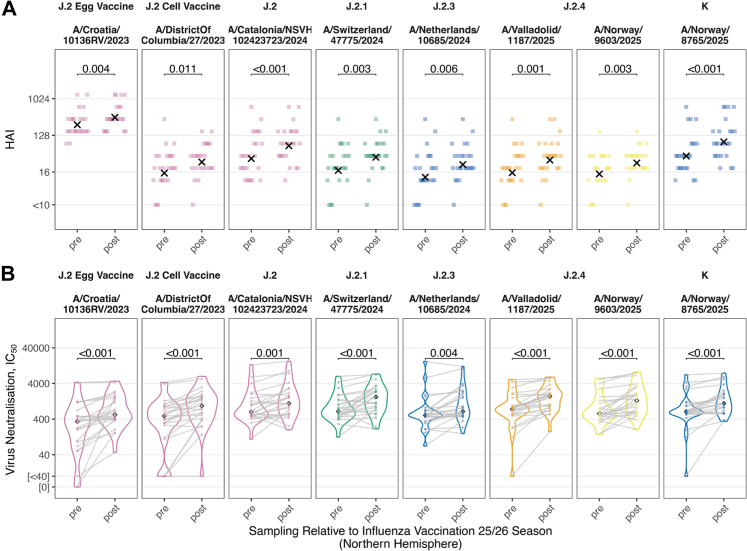
**Comparison of antibody titres against emergent Influenza A/H3N2 pre- and post-vaccination with a 2025/6 Northern Hemisphere inactivated egg-derived Trivalent Influenza Vaccine.** Distribution of antibody titres against Influenza A/H3N2 isolates quantified by haemagglutination inhibition (HAI) assay (**A**) and high-throughput live virus microneutralisation (MN) assay (**B**) in sera collected from the WIC cohort before (pre) and after (post) Influenza vaccination in the 2025/26 season. HAI are shown on a log_2_ scale with the geometric mean titre (×) and jittering along the x-axis. MN are shown as the IC_50_ on a log_10_ scale with the median titre (◊) where IC_50_ = dilution concentration of samples to achieve 50% inhibition of viral infection at 24 h. P values shown are from paired, two-tailed Wilcoxon signed rank tests.

Pre- and post-vaccination sera for individuals in the UK that received the 2025/26 Northern Hemisphere inactivated eTIV (containing A/Croatia/10136RV/2023) showed a boost of antibody titres to levels above the accepted 50% protective threshold of 40 HAI units^32^ for all A/H3N2 viruses tested across the majority of participants (Fig. 2A, Table S2). MN titres also showed significant boosting across all viruses (Fig. 2B, Table S3). Post-vaccination sera for individuals in Norway resulted in similar titres for the vaccine strains as well as the subclade K virus tested (Figure S1A and B, Table S6). These data indicate that even for the antigenically distinct J.2.3 and J.2.4/K groups, vaccination is as effective as against the vaccine strain, and importantly contrasts with the assessment of antisera generated from a single primary infection in ferrets (Fig. 1C and D). Of all the virus groups tested, the J.2.3 viruses showed on average the lowest recognition by the Northern Hemisphere vaccine in both HAI and MN assays (Fig. 2A and B, Tables S2 and S3). Although the K viruses have predominated in the 2025/26 Northern Hemisphere influenza season, the J.2.3 viruses do still circulate in some regions, particularly in South America and in some European countries, and vigilance is warranted should these viruses continue to evolve.

### Serological antibody profiling of cohort responses to recent A/H3N2 viruses: age-risk groups

**Fig. 3:**
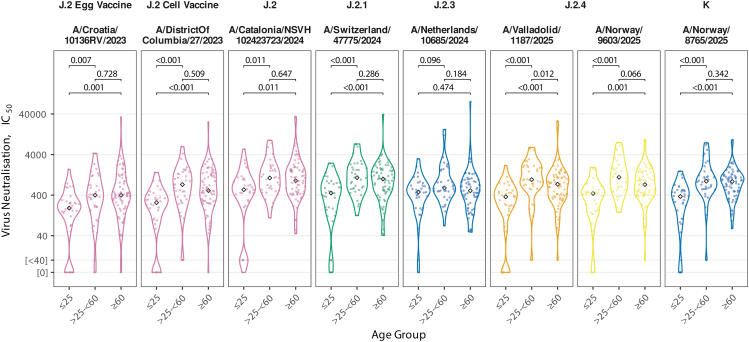
**Comparison of pre-vaccination serum neutralising antibodies against emergent Influenza A/H3N2 across age-risk groups across three cohorts.** Distribution of neutralising antibody titres against Influenza A/H3N2 2025 isolates quantified by high-throughput live virus microneutralisation (MN) assay in sera collected prior to influenza vaccination in 2025/26. MN are shown as the IC_50_ on a log_10_ scale with the median titre (◊) where IC_50_ = dilution concentration of samples to achieve 50% inhibition of viral infection at 24 h. P values shown are from Wilcoxon rank sum tests.

We next considered whether there was a large age-related effect to identify any at-risk groups. Stratifying our datasets by age-risk groups for influenza showed that MN titres increased with age group, with a significant monotonic trend across the three groups for all variants (Jonckheere–Terpstra test, p = <0.05), with the exception of A/Netherlands/10685/2024 (Jonckheere–Terpstra test, p = <0.87) (Fig. 3, Table S4 and S5). This trend applied to the majority of strains tested, suggesting that none of the age-risk groups had an immunity gap that the K clade viruses could exploit. The small reduction of recognition by the under 25 group suggests that fewer cumulative influenza exposures (exacerbated during the COVID-19 pandemic) likely reduced this group's antibody levels relative to the older groups, which have accrued more lifetime immunological experience (Fig. 3). Neutralising titres against the K subclade in the youngest age group tested (≤5 years) were also minimally different from older age groups in the NIPH cohort (Figure S1C, Table S7).

## Discussion

Seasonal influenza continues to cause significant public health burden, morbidity and mortality globally. Current mitigation relies on vaccine to reduce severe disease, particularly in the elderly, who are disproportionately at risk in an A/H3N2 predominant season.^33^ Although surveillance is undertaken continually, the punctuated timeline for vaccine recommendation and subsequent policy implementation means that there is inherent risk that an emerging strain might arise at a portion of “antigenic space” where the protective immunological profile of at-risk groups might be lowest. Additionally, the vaccine strain recommendation might not be antigenically similar to emerging viruses and may fail to afford protection. Our data suggest that despite the early arrival and near total predominance of the subclade K viruses in the early part of the 2025–26 influenza season in UK and Norway, the observed epidemiological dynamics were not a result of a fitness advantage the virus exploited when faced with population immunity.

We found that antibody titres induced by the 2025/26 inactivated eTIV, measured by two different modalities, were robust against all circulating groups of A/H3N2 viruses, demonstrating that despite genetic and antigenic heterogeneity, seasonal vaccines are still a safe and effective tool against divergent viruses.^4^^,^^7^^,^^34^ However, continued vigilance is warranted, particularly of groups of viruses that might circulate at lower levels but either rapidly evolve antigenically or recrudesce in another geographic region to predominate the following season. Importantly, evaluating sera from antigen-experienced participants provides a complementary perspective on risk compared with traditional antigenic mapping based on single-infection antisera.^35^ While antisera-based mapping can define a reasonable “worst-case” scenario, analyses using immune-experienced human populations, whose prior exposure, infection history, and vaccination status vary, can help determine how closely real-world immunity aligns with or diverges from that scenario. Together, these approaches offer complementary insights for interpreting potential risk.

If significantly lower neutralising antibody levels were indicated against the K subclade virus in a particular age-stratified group there might be observed effects such as increased hospitalisation. We carried out this particular analysis because individuals in younger age groups might have fewer prior influenza exposures conferring protection than older individuals. We observed that the under 25 age group did indeed have slightly lower titres, but this was true for all strains tested, suggesting that this alone could not account for the sudden rise of the subclade K viruses.

Using two distinct assay types in this study allowed for rapid, robust analyses and comparison of a varied sample set. We previously demonstrated a strong correlation between HAI titres and MN titres, validating our newly developed high-throughput MN assay for studying serum immunity to influenza viruses.^17^ The influenza MN assay builds on our SARS-CoV-2 assay, which, throughout the COVID-19 pandemic, had provided a method for near real-time assessment of predicted immunity to continually emerging variants. The same principle extends here in allowing us to determine quickly the threat level of emerging influenza viruses in different cohorts. Although we have shown strong correlation between the titres obtained from the HAI and MN assays, it is important to consider the differences between them and type of immunity being demonstrated in the results: MN titres will include functional neutralisation, whereas HAI does not detect NA or HA-stalk directed antibodies. In fact, our data shows that HAI might be detecting non-neutralising binding antibodies, as seen in the significantly higher HAI titres against the J.2 egg-based vaccine strain (A/Croatia/10136RV/2023) versus the other H3N2 strains tested (Fig. 2A), which was also observed by others,^4^ whereas we did not see this difference when we tested our samples using the MN assay.

Despite an early and dominant circulation of the subclade K viruses in the UK, the 2025–26 influenza season did not result in a substantially higher overall disease burden than recent years, with hospitalisation and laboratory confirmed case metrics remaining within expected seasonal ranges.^6^ Our MN data mirrored this real-world observation, showing that both pre-existing and vaccine-induced immunity offered comparable neutralising coverage across the A/H3N2 viruses tested.^34^ The MN assay ultimately offers increased data resolution and a more detailed assessment of functional neutralising antibody responses. This further demonstrates the value of the high-throughput influenza MN assay as an important tool for epidemic risk, policy, and surveillance assessments.

Our study provides a thorough assessment of the serological antibody landscape against emerging subclade K A/H3N2 Influenza viruses, but a few factors limit the full interpretation of the data. Our cohorts were diverse in age, but limited to the Northern European region, limiting applicability of these data to other geographical settings with different vaccination access and patterns of influenza circulation. And as with most human cohort studies, it is not possible to control for all possible representations and confounders: the vaccinated sub-cohort does not encompass all age groups (notably paediatrics) and vaccine types, and we are not able to assess effects of repeated vaccination since full vaccination histories were not collected. It could very well be that the baseline neutralisation and vaccine boost we see against the subclade K virus not seen in ferret antisera is due to prior vaccination or exposures. The Norway cohort analysis utilises residual serum samples collected through the public health system, which may introduce a bias toward individuals with poorer health status. This may better represent the population targeted by annual vaccination campaigns which are usually the elderly, those with comorbidities, and immune compromised patients. Despite this, they showed similar patterns of recognition to the UK-derived cohorts. Direct comparison between these two settings could not be carried out as these participants were not matched (only post-vaccination and residual samples) and the data were characterised using different assays (Supplementary Methods), but these data do serve to corroborate the findings across cohorts and demonstrate similarity when serological profiles are assessed by HAI and MN.

Our data support the integration of human immunogenicity assessments into seasonal influenza monitoring and epidemic risk assessments to accurately profile immune and vaccine escape, and to identify potential risk groups.

## Contributors

Conceptualisation: RH, DG, BW, MG, MYW, NSL, ECW, EF, OH, KB, LI.

Data curation: RH, AB, DG, CW, ECW, NSL, AR, YWC, JB, HWG, HW, KEB, AJS, AA, GA.

Formal analysis: CW, DG, AB, PSL, RH, MG, ECW, NSL, ELV, EF, AR, MYW.

Funding acquisition: ECW, NSL.

Investigation: RH, CW, DG, AB, MG, PSL, MG, MYW, ELV, EF, OH, CSP, LI.

Methodology: NSL, ECW, DG, AB, RH, MYW, ELV.

Project administration: MYW, RH, ECW, NSL, EF.

Resources: NSL, ECW, KB, MYW, SG, SG, CSP, LI.

Software: CW, DG, AB.

Supervision: RH, MG, EJC, MYW, ECW, NSL, KB, OH, EF, SG, SG, CSW, BW.

Validation: DG, CW, AB, PSL, RH, MG, EJC, MYW, ECW, NSL.

Visualisation: CW, DG, MG, AB.

Writing—original draft: NSL, RH, ECW, MYW.

Writing—review & editing: All authors including EJC, DB, CSP, LI.

MYW, ECW, NSL have accessed and verified the underlying data.

All authors have read and approved the final version of the manuscript.

## Data sharing statement

Anonymised data and R code for this research will be made freely available via github upon publication: https://github.com/FrancisCrickInstitute/ah3n2_analysis_public.

## Declaration of interests

ECW received consultancy fees from CSL Seqirus and served as vice-chair on the European Study Group for Infections of the Brain. BW received part salary support and was serving as director of the NIHR UCL Hospitals Biomedical Research Centre when this work was initiated. All other authors declare that they have no competing interests related to this work.
