# Supplementary material for: Assessment of human immunity to A/H3N2 influenza subclade K during 2025 emergence
Source: eBioMedicine. 2026 Jun 11;129:106332. doi: 10.1016/j.ebiom.2026.106332 (PMC13276524; doi:10.1016/j.ebiom.2026.106332)
Supplement: Supplementary Material [file mmc1.docx]

# **Supplemental Materials**

**Assessment of human immunity to A/H3N2 influenza subclade K during 2025 emergence**

Ruth Harvey^1^*, Ceilidh Welsh^2^*, Alexander M P Byrne^1^*, David Greenwood^2^*, Phoebe Stevenson-Leggett^3^*, Monica Galiano^1^, Dalan Bailey^4^, Yung-Wai Chan^5^, Jacob Boateng^5^, Hannah Wolmuth-Gordon^5^, Heather Whitaker^6^, Kevin E Brown^5^, Anna Jeffery-Smith^5^, Alex Allen^5^, Gayatri Amirthanlingam^5^, Even Fossum^7,8^, Elisabeth Lea Vikse^7,8^, Andreas Rohringer^7,8^, Olav Hungnes^7,8^, Karoline Bragstad^7,8^, Charles Swanton^2,9^, Bryan Williams^9,11^, Sonia Gandhi^2,9^, Steve Gamblin^2^, Edward J Carr^2,10^, Nicola S Lewis^1,13^* Emma C Wall^2,11,12^*^corr^, and Mary Y Wu^3^*^corr^, on behalf of the Crick Serology Pipeline and Legacy Investigators

1. Worldwide Influenza Centre, Francis Crick Institute, London, UK
2. Francis Crick Institute, London, UK
3. Viral & Immune Surveillance Platform, Francis Crick Institute, UK
4. The Pirbright Institute, Ash Road, Pirbright, UK
5. Immunisation and Vaccine Preventable Diseases Division, UK Health Security Agency, London, UK
6. Statistics, Modelling and Economics Department, UK Health Security Agency, London, UK
7. National influenza Centre, Oslo, Norway
8. Department of Virology, Division of Infection Control, Norwegian Institute of Public Health, Oslo, Norway
9. University College London, UK
10. UCL Centre for Renal and Bladder Health, University College London, UK
11. National Institute for Health Research Biomedical Research Centre, University College London Hospitals, London, UK
12. Centre for Infection and Immunobiology, Blizard Institute, Queen Mary University of London, UK
13. Royal Veterinary College, Royal College Street, London, UK

*These authors contributed equally

Correspondence to [emma.wall@crick.ac.uk](mailto:emma.wall@crick.ac.uk) and [mary.wu@crick.ac.uk](mailto:mary.wu@crick.ac.uk)

Keywords: Seasonal Influenza; H3N2, Subclade K, human serology; antigenic cartography; vaccine; neutralising antibodies, haemagglutination inhibition

**Consortium authorship**

**Crick Serology Pipeline:**

| **First Names** | **Surnames** |
| --- | --- |
| Giulia | Dowgier |
| Agnieszka | Hobbs |
| Choon Ping | Tan |
| Jarod | Zvartau-Hind |
| Murad | Miah |
| Odiesia | Daley |
| Mauro | Miranda |
| Philip | Bawumia |
| Nicola | O’Reilly |
| Chris | Cheshire |
| Nicholas | Wilson |
| Karen | Ambrose |
| Amy | Strange |
| Gavin | Kelly |
| Svend | Kjær |

**Legacy Investigators:**

| **First Names** | **Surnames** |
| --- | --- |
| Rupert CL | Beale |
| Padmasavee | Papineni |
| Tumena | Corrah |
| Richard | Gilson |

**Supplemental figures**

**Figure S1**

**Figure S1: Comparison of antibody titres against emergent Influenza A/H3N2 in Norwegian Institute of Public Health (NIPH) immunised and residual sera.** Distribution of neutralising antibody titres against Influenza A/H3N2 2025 isolates quantified by haemagglutination assay (**A; supplemental methods**) and low-throughput live virus microneutralisation (MN) assay (**B, C; supplemental methods**) in sera collected after (post) Influenza vaccination in healthy adults in 2025/26 (**A, B**). (**C**) Neutralising titres in anonymised residual sera collected in August 2025 by age groups. HAI and low-throughput MN are shown on a log2 scale with the geometric mean titre (x).

**Figure S2**

**Figure S2: Assessment of bootstrapped antigen and serum positions for antigenic cartography of A/H3N2 strains.** Antigenic cartography of A/H3N2 strains generated from haemagglutination inhibition (HAI; **A**) and microneutralisation (MN; **B**) assay data obtained with ferret antisera. Antigen and serum positions are bootstrapped 1000 times using noisy bootstrapping method, with a 68% confidence level. In both A and B, strains are coloured by subclade and labelled with associated strain.

**Supplemental tables**

| **Table S1: Summary of viruses used in the analysis**   \| **H3N2 viruses evaluated** \| **Clade** \| **Additional HA substitutions** \| \| --- \| --- \| --- \| \| A/Croatia/10136RV/2023 \| J.2 Egg Vaccine \|  \| \| A/DistrictOfColumbia/27/2023 \| J.2 Cell Vaccine \|  \| \| A/Catalonia/NSVH102423723/202 \| J.2 \| HA1: T135K \| \| A/Switzerland/47775/2024 \| J.2.1 \|  \| \| A/Netherlands/10685/2024 \| J.2.3 \|  \| \| A/Valladolid/1187/2025 \| J.2.4 \|  \| \| A/Norway/09603/2025 \| J.2.4 \| HA1: F79V,S144N,N158D,I160K,T328A \| \| A/Norway/8765/2025 \| K \|  \| |
| --- | --- | --- | --- | --- | --- | --- | --- | --- | --- | --- | --- | --- | --- | --- | --- | --- | --- | --- | --- | --- | --- | --- | --- | --- | --- | --- | --- |

**Table S2: Geometric mean titre (GMT) and paired fold-changes (FC) in Haemaglutination Inhibition (HAI) induced by Influenza Vaccination in 2025/26**

|  |  | **HAI, GMT (Geometric SD)** | |  |  |
| --- | --- | --- | --- | --- | --- |
| **Strain** | **Clade** | **Pre-vaccine** | **Post-vaccine** | **FC (95%**  **Confidence interval)** | **P** |
| A/Croatia/10136RV/2023 | J.2 Egg Vaccine | 242.51 (2.06) | 367.58 (2) | 1.52 (1.25-1.95) | 0.004 |
| A/DistrictOfColumbia/27/2023 | J.2 Cell Vaccine | 19.45 (3.07) | 32.94 (2.53) | 1.52 (1.28-1.95) | 0.011 |
| A/Catalonia/NSVH102423723/2024 | J.2 | 41.12 (3.35) | 80 (2.83) | 1.95 (1.56-2.57) | <0.001 |
| A/Switzerland/47775/2024 | J.2.1 | 22.97 (3.16) | 43.47 (2.57) | 1.69 (1.36-2.31) | 0.003 |
| A/Netherlands/10685/2024 | J.2.3 | 16.02 (3.42) | 28.68 (2.67) | 1.52 (1.21-1.96) | 0.006 |
| A/Valladolid/1187/2025 | J.2.4 | 20 (3.16) | 37.84 (2.88) | 1.69 (1.39-2.17) | 0.001 |
| A/Norway/9603/2025 | J.2.4 | 17.9 (2.7) | 30.31 (2.23) | 1.52 (1.25-1.89) | 0.003 |
| A/Norway/8765/2025 | K | 45.95 (3.1) | 99.87 (2.98) | 2.17 (1.69-3.29) | <0.001 |

**Table S3: Median high-throughput live virus microneutralisation (MN) assay titres and paired fold-changes (FC) induced by Influenza Vaccination in 2025/26**

|  |  | **MN, Median [IQR] IC50** | |  |  |
| --- | --- | --- | --- | --- | --- |
| **Strain** | **Clade** | **Pre-vaccine** | **Post-vaccine** | **FC (95% Confidence interval)** | **P** |
| A/Croatia/10136RV/2023 | J.2 Egg Vaccine | 344.15  [92.62-488.39] | 527.56 [392.43-1532.21] | 1.82 (1.34-3.29) | <0.001 |
| A/DistrictOfColumbia/27/2023 | J.2 Cell Vaccine | 488.39  [267.14-891.86] | 932.44 [417.6-1727.12] | 2.04 (1.46-3.3) | <0.001 |
| A/Catalonia/NSVH102423723/2024 | J.2 | 637.06  [495.65-1357.94] | 1101.57 [568.76-2330.88] | 1.49 (1.08-1.86) | 0.001 |
| A/Switzerland/47775/2024 | J.2.1 | 653.55  [487.27-1322.24] | 1666.56 [789.39-2453.8] | 1.56 (1.3-1.77) | <0.001 |
| A/Netherlands/10685/2024 | J.2.3 | 509.06  [450.57-705.16] | 654.22 [495.76-2074.11] | 1.35 (1.1-1.87) | 0.004 |
| A/Valladolid/1187/2025 | J.2.4 | 762.18  [527.91-1133.1] | 1757.47 [906.53-2290.97] | 1.81 (1.33-2.43) | <0.001 |
| A/Norway/9603/2025 | J.2.4 | 573.14  [446.78-1157.87] | 1322.4 [614.16-2443.54] | 1.57 (1.26-2.35) | <0.001 |
| A/Norway/8765/2025 | K | 646.07  [554.34-968.94] | 1095.98 [587.19-2059.83] | 1.69 (1.23-1.82) | <0.001 |

**Table S4: Fold-changes (FC) in high-throughput live virus microneutralisation (MN) assay titres against A/H3N2 strains by age-risk group**

|  |  |  | **MN, Median IC50** |  |  |  |
| --- | --- | --- | --- | --- | --- | --- |
| **Strain** | **Clade** | **Comparison** | **Group2/Group1** | **FC (95% Confidence Interval)** | **P** | **Adj. P** |
| A/Catalonia/NSVH102423723/2024 | J.2 | >25-<60/≤25 | 1061.84/547.05 | 1.9 (1.09-3.11) | 0.005 | 0.011 |
| A/Croatia/10136RV/2023 | J.2 Egg Vaccine | >25-<60/≤25 | 399.72/191.33 | 2 (1.17-3.03) | 0.005 | 0.007 |
| A/DistrictOfColumbia/27/2023 | J.2 Cell Vaccine | >25-<60/≤25 | 727.89/262 | 3.16 (1.71-6.37) | <0.001 | <0.001 |
| A/Netherlands/10685/2024 | J.2.3 | >25-<60/≤25 | 602.23/475.36 | 1.28 (0.92-1.85) | 0.032 | 0.096 |
| A/Norway/8765/2025 | K | >25-<60/≤25 | 881.58/370.95 | 2.64 (1.49-4.39) | <0.001 | <0.001 |
| A/Norway/9603/2025 | J.2.4 | >25-<60/≤25 | 1103.58/440.82 | 2.8 (1.44-4.68) | <0.001 | <0.001 |
| A/Switzerland/47775/2024 | J.2.1 | >25-<60/≤25 | 1077.25/451.38 | 2.42 (1.1-4.54) | <0.001 | <0.001 |
| A/Valladolid/1187/2025 | J.2.4 | >25-<60/≤25 | 963.66/363.38 | 3.43 (1.96-7.25) | <0.001 | <0.001 |
| A/Catalonia/NSVH102423723/2024 | J.2 | ≥60/≤25 | 904.26/547.05 | 1.88 (1.08-2.53) | 0.007 | 0.011 |
| A/Croatia/10136RV/2023 | J.2 Egg Vaccine | ≥60/≤25 | 395.6/191.33 | 2.15 (1.55-2.78) | <0.001 | 0.001 |
| A/DistrictOfColumbia/27/2023 | J.2 Cell Vaccine | ≥60/≤25 | 518.53/262 | 2.2 (1.51-3.64) | <0.001 | <0.001 |
| A/Netherlands/10685/2024 | J.2.3 | ≥60/≤25 | 514.96/475.36 | 1.13 (0.77-1.55) | 0.474 | 0.474 |
| A/Norway/8765/2025 | K | ≥60/≤25 | 866.55/370.95 | 2.33 (1.43-3.79) | <0.001 | <0.001 |
| A/Norway/9603/2025 | J.2.4 | ≥60/≤25 | 728.26/440.82 | 1.84 (1.12-3.06) | <0.001 | 0.001 |
| A/Switzerland/47775/2024 | J.2.1 | ≥60/≤25 | 1000.69/451.38 | 2.22 (1.38-3.59) | <0.001 | <0.001 |
| A/Valladolid/1187/2025 | J.2.4 | ≥60/≤25 | 742.65/363.38 | 2.25 (1.37-3.56) | <0.001 | <0.001 |
| A/Catalonia/NSVH102423723/2024 | J.2 | ≥60/>25-<60 | 904.26/1061.84 | 1.05 (0.6-1.61) | 0.647 | 0.647 |
| A/Croatia/10136RV/2023 | J.2 Egg Vaccine | ≥60/>25-<60 | 395.6/399.72 | 1.06 (0.73-1.81) | 0.728 | 0.728 |
| A/DistrictOfColumbia/27/2023 | J.2 Cell Vaccine | ≥60/>25-<60 | 518.53/727.89 | 0.83 (0.5-1.44) | 0.509 | 0.509 |
| A/Netherlands/10685/2024 | J.2.3 | ≥60/>25-<60 | 514.96/602.23 | 0.87 (0.62-1.23) | 0.123 | 0.184 |
| A/Norway/8765/2025 | K | ≥60/>25-<60 | 866.55/881.58 | 0.97 (0.64-1.38) | 0.342 | 0.342 |
| A/Norway/9603/2025 | J.2.4 | ≥60/>25-<60 | 728.26/1103.58 | 0.72 (0.42-1.13) | 0.066 | 0.066 |
| A/Switzerland/47775/2024 | J.2.1 | ≥60/>25-<60 | 1000.69/1077.25 | 0.93 (0.5-1.58) | 0.286 | 0.286 |
| A/Valladolid/1187/2025 | J.2.4 | ≥60/>25-<60 | 742.65/963.66 | 0.68 (0.3-1.02) | 0.012 | 0.012 |

**Table S5: Median and Interquartile Range (IQR) for high-throughput live virus microneutralisation (MN) assay titres against A/H3N2 strains by age-risk group**

| **Strain** | **Age Group** | **Median** | **IQR** |
| --- | --- | --- | --- |
| A/Croatia/10136RV/2023 | ≤25 | 191.33 | 84.55-274.27 |
| A/Croatia/10136RV/2023 | >25-<60 | 399.72 | 194.07-844.22 |
| A/Croatia/10136RV/2023 | ≥60 | 395.6 | 211.95-1066.11 |
| A/DistrictOfColumbia/27/2023 | ≤25 | 262 | 95.99-350.35 |
| A/DistrictOfColumbia/27/2023 | >25-<60 | 727.89 | 310.18-1373.9 |
| A/DistrictOfColumbia/27/2023 | ≥60 | 518.53 | 265-1337.56 |
| A/Catalonia/NSVH102423723/2024 | ≤25 | 547.05 | 284.95-972.42 |
| A/Catalonia/NSVH102423723/2024 | >25-<60 | 1061.84 | 539.68-1887.6 |
| A/Catalonia/NSVH102423723/2024 | ≥60 | 904.26 | 465.87-1755.19 |
| A/Switzerland/47775/2024 | ≤25 | 451.38 | 175.39-793.04 |
| A/Switzerland/47775/2024 | >25-<60 | 1077.25 | 573.19-2021.22 |
| A/Switzerland/47775/2024 | ≥60 | 1000.69 | 509.06-1653.19 |
| A/Netherlands/10685/2024 | ≤25 | 475.36 | 230.36-687.32 |
| A/Netherlands/10685/2024 | >25-<60 | 602.23 | 495.19-1620.89 |
| A/Netherlands/10685/2024 | ≥60 | 514.96 | 254.82-963.38 |
| A/Valladolid/1187/2025 | ≤25 | 363.38 | 168.98-512.51 |
| A/Valladolid/1187/2025 | >25-<60 | 963.66 | 688.09-2014.83 |
| A/Valladolid/1187/2025 | ≥60 | 742.65 | 352.58-1134.5 |
| A/Norway/9603/2025 | ≤25 | 440.82 | 162.93-530.17 |
| A/Norway/9603/2025 | >25-<60 | 1103.58 | 531.84-2030.25 |
| A/Norway/9603/2025 | ≥60 | 728.26 | 413.3-1451.5 |
| A/Norway/8765/2025 | ≤25 | 370.95 | 120.5-532.14 |
| A/Norway/8765/2025 | >25-<60 | 881.58 | 566.44-1565.69 |
| A/Norway/8765/2025 | ≥60 | 866.55 | 485.03-1214.25 |

**Table S6: Haemaglutinin inhibition (HAI) and low-throughput live virus microneutralisation (MN) geometric mean titres (GMT) induced by Influenza Vaccination in 2025/26 in the Norwegian Institute of Public Health (NIPH) cohort (immunised)**

| **Strain** | **Clade** | **Assay** | **GMT post-vaccine** | **Geometric SD post-vaccine** | **Range** |
| --- | --- | --- | --- | --- | --- |
| A/Croatia/10136RV/2023 | J.2 Egg Vaccine | HAI | 88.33 | 2.95 | 20-1280 |
| A/DistrictOfColumbia/27/2023 | J.2 Cell Vaccine | HAI | 63.50 | 2.29 | 20-320 |
| A/Norway/8765/2025 | K | HAI | 61.43 | 2.43 | 10-320 |
| A/Croatia/10136RV/2023 | J.2 Egg Vaccine | MN | 100.79 | 1.88 | 40-320 |
| A/DistrictOfColumbia/27/2023 | J.2 Cell Vaccine | MN | 63.50 | 1.81 | 20-160 |
| A/Norway/8765/2025 | K | MN | 44.16 | 2.09 | 10-160 |

**Table S7: Geometric mean titres (GMT) of low-throughput live virus microneutralisation (MN) titres by age-group in the Norwegian Institute of Public Health (NIPH) cohort (residual)**

| **Strain** | **Clade** | **Assay** | **Age group** | **GMT pre-vaccine** | **Geometric SD pre-vaccine** | **Range** |
| --- | --- | --- | --- | --- | --- | --- |
| A/DistrictOfColumbia/27/2023 | J.2 Cell Vaccine | MN | ≤5 | 27.95 | 3.54 | 5-160 |
| A/DistrictOfColumbia/27/2023 | J.2 Cell Vaccine | MN | >5-≤15 | 40.00 | 3.19 | 5-320 |
| A/DistrictOfColumbia/27/2023 | J.2 Cell Vaccine | MN | ≥60 | 22.08 | 3.09 | 5-320 |
| A/Norway/8765/2025 | K | MN | ≤5 | 26.01 | 2.96 | 5-80 |
| A/Norway/8765/2025 | K | MN | >5-≤15 | 32.32 | 2.86 | 5-320 |
| A/Norway/8765/2025 | K | MN | ≥60 | 20.81 | 2.7 | 5-80 |

**Supplemental methods (for the Norwegian Institute of Public Health cohorts)**

**Virus propagation and titration**

The Influenza A/Croatia/10136RV/2023 and A/District of Columbia/27/2023 were provided by the UK WHO Collaborating Centre for Reference and Research on Influenza, the Worldwide Influenza Centre (WIC) at the Francis Crick Institute.

H3N2 influenza virus stocks were propagated in Madin-Darby canine kidney-α2.6-sial transferase (MDCK-SIAT1) cells (A/District of Columbia/27/2023 and A/Norway/8765/2025) and embryonated chicken eggs (A/Croatia/10136RV/2023). Virus stocks were titrated with MDCK-SIAT1 cells using ELISA to identify culture wells positive for virus growth. The 50% tissue culture infectious dose (TCID_50_) for each stock was calculated using Reed-Muench method (Reed & Muench, 1938).

**Low-throughput live virus microneutralisation assay (MNA)**

All serum samples were heat-inactivated at 56°C for 30 min and serially diluted two-fold in virus diluent (MEM supplemented with 0.5% bovine serum albumin fraction V, 2.5mM HEPES buffer, 1x penicillin/streptomycin and 1µg/ml TPCK-treated trypsin) in a 96-well culture plates. Virus inoculum corresponding to 200 TCID_50_ per 50µL was mixed with the diluted serum samples and incubated for 1 h at 37°C.

Following incubation, MDCK-SIAT1 cells were added at a density of 1.5 x 10^4^ cells per well, and plates were incubated for 20 h at 37°C. The plates were washed with PBS and then fixed with cold 80% acetone in PBS. Virus replication was assessed by ELISA.

The ELISA included a blocking step with PBS containing 1% BSA, primary incubation with anti-influenza A virus nucleoprotein (NP) monoclonal antibody (InVivoMab, BE0159), and secondary incubation with Goat anti-mouse IgG ALP Antibody (Sigma-Aldrich, A2429). Between each incubation step, the plates were washed with wash buffer (PBS with 2% Tween 20). Finally, 1 mg/mL of phosphatase substrate dissolved in diethanolamine buffer was added to the plates, and the absorbance was measured at 405 nm after 45 min.

The neutralisation end point titre was determined as the reciprocal of the highest serum dilution resulting in an OD value less than 2 times the mean of the cells-only controls. Sera with titres < 10 were assigned an HAI titre of 5.

**Hemagglutination inhibition assay (HAI)**

Aliquots of collected sera were treated with receptor destroying enzyme (RDE) overnight (ON) at 37°C, followed by the addition of 1.5% sodium citrate and inactivation by incubating for 30 min at 56°C. Next, the sera were serially diluted two-fold in 96-well plates starting at dilution 1:20 in PBS pH 7.2 containing 20nM oseltamivir, resulting in a final volume of 25 μl pr well. Viral antigens (A/Croatia/10136RV/2023, A/District of Columbia/27/2023 or A/Norway/8765/2025) were subsequently added to the wells at a concentration 6–8 hemagglutinating units (HAU) per 25 μl. Diluted sera and influenza virus were incubated for 1h at room temperature, before addition of turkey red blood cells (RBC) as indicator cells at a concentration of 0.25% in PBS with BSA. The HAI titre was determined as the serum dilution factor that produced complete inhibition in the assay. Sera with titres < 20 were assigned an HAI titre of 10.
